# CNPY3’s regulation of tumor microenvironment and its impact on colon cancer aggressiveness

**DOI:** 10.1186/s10020-025-01145-1

**Published:** 2025-03-07

**Authors:** Xucan Gao, Biaohuan Zhou, Xiudong Feng, Zhouxin Ji, Qiang Li, Huining Liu

**Affiliations:** 1 Department of Anorectal Surgery, Shenzhen People’s Hospital (The Second Clinical Medical College, Jinan University, The First Affiliated Hospital, Southern University of Science and Technology), Shenzhen, Guangdong 518020 China; 2 Department of Operating Room, Shenzhen People’s Hospital (The Second Clinical Medical College, Jinan University, The First Affiliated Hospital, Southern University of Science and Technology), Shenzhen, Guangdong 518020 China

**Keywords:** CNPY3, Colon cancer, Tumor microenvironment, Biomarker, Tumorigenesis

## Abstract

**Background:**

Canopy FGF signaling regulator 3 (CNPY3) has been implicated in tumor progression. However, its specific role in colon cancer (CC) remains unclear. This study aims to investigate the function of CNPY3 in CC and its potential as a therapeutic target.

**Methods:**

A total of 201 CC tissue specimens and 67 adjacent non-cancerous tissues were collected for analysis. CNPY3 expression was assessed using immunohistochemistry and quantitative real-time PCR. Functional assays were conducted in CC cell lines (HT-29 and SW-620) following CNPY3 knockdown to evaluate its effects on cell proliferation, migration, and apoptosis. Gene expression profiling, fibroblast co-culture experiments, and in vivo xenograft models were also conducted.

**Results:**

Increased CNPY3 expression correlated with advanced tumor stages and poorer prognosis. Knockdown of CNPY3 significantly inhibited cell proliferation, migration, and induced apoptosis in CC cell lines. CNPY3 depletion also modulated fibroblast behavior, inhibiting their transformation into cancer-associated fibroblasts. Pathway analysis revealed that CNPY3 knockdown affected the cell cycle and p53 signaling pathways, and reduced activation of the MAPK and PI3K/AKT pathways. Additionally, CNPY3 knockdown enhanced CC cell sensitivity to 5-fluorouracil. In vivo studies demonstrated that CNPY3 knockdown resulted in smaller tumor sizes and weights than controls.

**Conclusions:**

CNPY3 is a crucial regulator in CC progression, correlating with tumor aggressiveness and poor patient outcomes. Targeting CNPY3 may offer a promising therapeutic strategy and a valuable prognostic marker in CC management.

**Supplementary Information:**

The online version contains supplementary material available at 10.1186/s10020-025-01145-1.

## Background

Colon cancer (CC) is one of the leading causes of cancer-related mortality worldwide, marked by high recurrence and metastasis rates that complicate treatment efforts (Benson et al. 2024). The global burden of CC is substantial. It ranks as the third most commonly diagnosed cancer and the second leading cause of cancer death, according to World Health Organization statistics (Chalabi et al. 2024). Despite advances in early detection and treatment options, patients with advanced-stage CC have limited therapeutic options and poor prognoses (Nair et al. 2024). This highlights the urgent need for a deeper understanding of the molecular mechanisms driving CC pathogenesis and progression (Ballal and Saklani 2024).

Research has shown that CC development involves a complex interplay of genetic mutations, such as epigenetic modifications (Tatsumi 2024), environmental factors, and cellular interactions within the tumor microenvironment (Wu et al. 2024). Studies have identified critical pathway alterations, such as Wnt/β-catenin (Liu et al. 2024), PI3K/AKT, and MAPK signaling pathways (Wang et al. 2022). However, beyond these well-studied mechanisms, the role of the tumor microenvironment in supporting cancer progression is still being unraveled (Malla et al. 2022). Cancer-associated fibroblasts (CAFs), in particular, have emerged as pivotal players in this microenvironment (Strating et al. 2023). CAFs support tumor growth, invasion, and immune evasion through the secretion of growth factors, cytokines, and extracellular matrix components that create an immunosuppressive environment conducive to tumor expansion (Chen et al. 2024a, b, c). Targeting CAFs or their signaling pathways may offer new therapeutic strategies to disrupt these pro-tumorigenic interactions (Agorku et al. 2024).

Amid the search for molecules that play roles in CC progression, CNPY3 (Canopy FGF signaling regulator 3), a member of the canopy protein family, has attracted research interest (Zhang et al. 2024). CNPY3 is implicated in diverse cellular processes, such as cell cycle regulation and signal transduction (Ghait et al. 2022). Notably, high CNPY3 expression has been observed in several malignancies, including gastric and prostate cancers, where it is associated with aggressive tumor characteristics and poor prognosis (Zhang et al. 2024; Zhou et al. 2023). These findings, coupled with the upregulation of CNPY3 in colon adenocarcinoma from our previous study, support its relevance in cancer biology, particularly through its involvement in key pathways like PI3K/AKT signaling. This pathway is crucial in promoting tumorigenic behaviors, including cell proliferation and survival, which are hallmarks of cancer aggressiveness and treatment resistance (Gao et al. 2024). Given its established role in oncogenic pathways (Stefani et al. 2021), it stands to reason that CNPY3 may also influence signaling pathways between tumor cells and the surrounding stromal cells (Wei et al. 2024a, b). Notably, CAFs play a key role in remodeling the tumor microenvironment by secreting factors that support cancer progression (Cords et al. 2024), making it essential to investigate whether CNPY3 regulates fibroblast behavior and contributes to CAF activation.

This study focuses on investigating the expression patterns of CNPY3 in CC tissues and cell lines, examining its correlations with clinical parameters and patients’ prognosis. Through techniques such as immunohistochemistry, we aim to assess whether increased CNPY3 expression correlates with disease severity or unfavorable clinical outcomes. Using gene knockdown experiments in CC cell lines, we further explore the functional roles of CNPY3 in cell proliferation, migration, apoptosis, and cell cycle. Additionally, given the pivotal role of CAFs in CC, we examine whether CNPY3 influences fibroblast behaviors, particularly their proliferation, migration, and transformation into CAFs. By elucidating the molecular pathways through which CNPY3 impacts tumor and stromal cell interactions, this research aims to provide a foundation for new therapeutic approaches that disrupt supportive microenvironment interactions in CC.

## Methods

### Collection of clinical specimens

Between January 2014 and March 2018, a total of 201 tissue specimens from colon cancer (CC) and 67 adjacent non-cancerous tissues were collected from patients at Shenzhen People’s Hospital. Ethical approval was obtained from the hospital’s Ethics Committee. Written informed consent was received from each patient. Tissue samples for RNA analysis were promptly frozen in liquid nitrogen and stored at -80 °C, while samples for immunohistochemical analysis were fixed in 4% paraformaldehyde. Patients included in the study had histologically confirmed CC and had undergone resection with the intent of curing the disease. Patients with rectal cancer, prior neoadjuvant therapy, or non-curative or emergency procedures were excluded from the analysis.

### Immunohistochemical (IHC) staining

Tissue microarrays were assembled and subjected to immunohistochemical staining following established protocols. Primary antibodies against CNPY3 (1:200 dilution, Proteintech) and Ki67 (1:500 dilution, Abcam) were applied were applied, followed by secondary antibodies conjugated to horseradish peroxidase (Abcam). Tissue slides were deparaffinized, rehydrated, and blocked before being incubated with the primary antibody at 4 °C overnight. DAB (diaminobenzidine) was used for visualization, and two pathologists independently evaluated staining results. Immunohistochemistry scores (IHS) were assigned based on staining intensity (scored from 0 to 3) and the percentage of positive cells (0–1), producing a total score between 0 and 3.

### Bioinformatics assessment

Gene expression levels of CNPY3 in colon adenocarcinoma (COAD) were analyzed using data from The Cancer Genome Atlas (TCGA), with the Gene Expression Profiling Interactive Analysis (GEPIA) platform used for visualization. Logarithmic transformation was applied to expression data for enhanced clarity and graphical presentation. Differently expressed genes between normal and CNPY3-knockdown HT-29 cells were identified with the “limma” package. Functional annotation was conducted using Gene Ontology (GO) and Kyoto Encyclopedia of Genes and Genomes (KEGG) analysis.

### Quantitative real-time PCR (qRT-PCR) analysis

RNA was extracted with TRIzol, then converted to complementary DNA (cDNA), and qRT-PCR was performed on an ABI 7900HT using SYBR Green dye. Conditions included initial denaturation at 95 °C for 10 min, followed by 45 cycles at 95 °C for 15 s and 60 °C for 34 s. Relative expression of CNPY3 was calculated using the 2^^−ΔΔCT^ method. Primer sequences can be found in Supplementary Table 1.

### Prognostic evaluation

Patients were stratified into high and low CNPY3 expression groups based on qRT-PCR results. Kaplan-Meier survival curves were generated to evaluate differences in overall survival (OS) between groups. The predictive strength of CNPY3 expression was assessed with ROC curve analysis, where an area under the curve (AUC) above 0.7 was considered indicative of strong predictive value. Cox proportional hazard models, both univariate and multivariate, were applied to determine CNPY3’s impact on prognosis.

### Cell culture and genetic manipulation

Human colonic epithelial cell lines (HCoEpiC, NCM460), CC lines (T84, DLD1, Caco-2, HCT-116, HT-29, SW-48, SW-480, SW-620), and fibroblasts (CCD-18Co) were obtained from ATCC or the Chinese Academy of Sciences. Cells were grown in DMEM with 10% FBS. Lentiviral constructs for CNPY3 knockdown were designed by GenePharma (Shanghai, China), and stable knockdown lines were generated post-transduction.

### Cell proliferation measurement

The CCK-8 assay quantified cell proliferation. Cells were seeded in 96-well plates, with viability measured at 0, 24, 48, 72, and 96 h by reading absorbance at 450 nm. For drug sensitivity analysis, after overnight culture, 5-FU was added to the cells at final concentrations of 0, 50, 100, 200, 400, 600, and 800 µg/mL. All cells were then cultured for 72 h and the viability was measured. A concentration-effect curve was plotted with drug concentration on the x-axis and relative cell viability on the y-axis, allowing for the calculation of half-maximal inhibitory concentration (IC50) values.

### Wound healing assay

Cells were cultured to confluence in 6-well plates, and a standardized scratch was made using a pipette tip. Images were captured at 0, 6, and 12 h with Hybrid Cell Count software (BZ-X810, Keyence), and migration was quantified based on the percentage of wound closure.

### Flow cytometry for apoptosis and cell cycle

To assess apoptosis, cells were labeled with FITC-Annexin V and propidium iodide. For cell cycle analysis, cells were fixed, permeabilized, and stained with propidium iodide. Data was gathered on a BD FACS system to analyze apoptotic rates and cell cycle phases.

### Western blot analysis

Cell lysates were prepared with RIPA buffer containing protease and phosphatase inhibitors to detect CNPY3, PI3K, AKT, ERK, JNK, and phosphorylated forms. Protein quantification was done with a BCA assay, loading 20–30 µg per sample onto SDS-PAGE gels. Proteins were transferred to PVDF membranes, blocked with 5% non-fat milk, and incubated overnight with primary antibodies. HRP-conjugated secondary antibodies were applied, and visualization was achieved with enhanced chemiluminescence (ECL) imaging.

### Microarray analysis

Quality RNA was extracted and validated on an Agilent 2200 TapeStation. Purified cDNA was synthesized and hybridized to a Human Clariom S Array. Hybridized arrays were rinsed and scanned on an Affymetrix GeneChip Scanner 3000 7G.

### Fibroblast migration assay

The migration capacity of fibroblasts was assessed using cell culture inserts with an 8 μm pore size. After 72 h of transfection, CCD-18Co cells were placed in the upper chamber, with HT-29 or SW-620 cells in the lower chamber. Migration was visualized with Diff-Quick stain.

### ELISA for secreted factors

After 72 h, 1.2 × 10^^5^ cells per well were seeded in six-well plates for five days. ELISA kits (Abcam) measured TGFB1, PDGF-BB, EGFR, and VEGF in culture supernatants per kit protocols (Notodihardjo et al. 2019).

### Fibroblast and CC cell Co-Culture

Seventy-two hours post-transfection, HT-29 and SW-620 cells were co-cultured with CCD-18Co fibroblasts. Cells were seeded into co-culture inserts with 0.4 μm pores and incubated at 37 °C with 5% CO_2_ for five days.

### Xenograft model experiments

In vivo studies were conducted with Shenzhen People’s Hospital Ethics Committee approval. Six-week-old BALB/c nude mice were injected subcutaneously with 5 × 10^^6^ transfected HT-29 cells. For in vivo 5-FU sensitivity analysis, ten days after cancer cell implantation, mice were injected intraperitoneally with 5-FU (30 mg/kg) or phosphate buffer saline (PBS) every 3 days for six cycles. Thus, the mice were divided into four groups: KD-Con + PBS, KD + PBS, KD-Con + 5-FU, and KD + 5-FU. Tumor growth was monitored by measuring the tumor volumes every week, and the mice were euthanized at the end of the study for histological and molecular analysis. Tumor tissues were harvested for further investigation, including Ki67 immunohistochemistry, to assess cell proliferation and evaluate the effects of CNPY3 knockdown and 5-FU treatment on tumor progression and therapeutic response.

### Statistical analysis

Data analysis was performed using R 4.2.1 and SPSS 26.0. Statistical significance was set at *P* < 0.05, with Student’s t-test and χ^2^ tests for continuous and categorical data, respectively. Cox regression, Kaplan-Meier, and ROC curve analysis were used for prognostic evaluations.

## Results

### Elevated CNPY3 expression observed in colon cancer tissues and cell lines

**Fig. 1:**
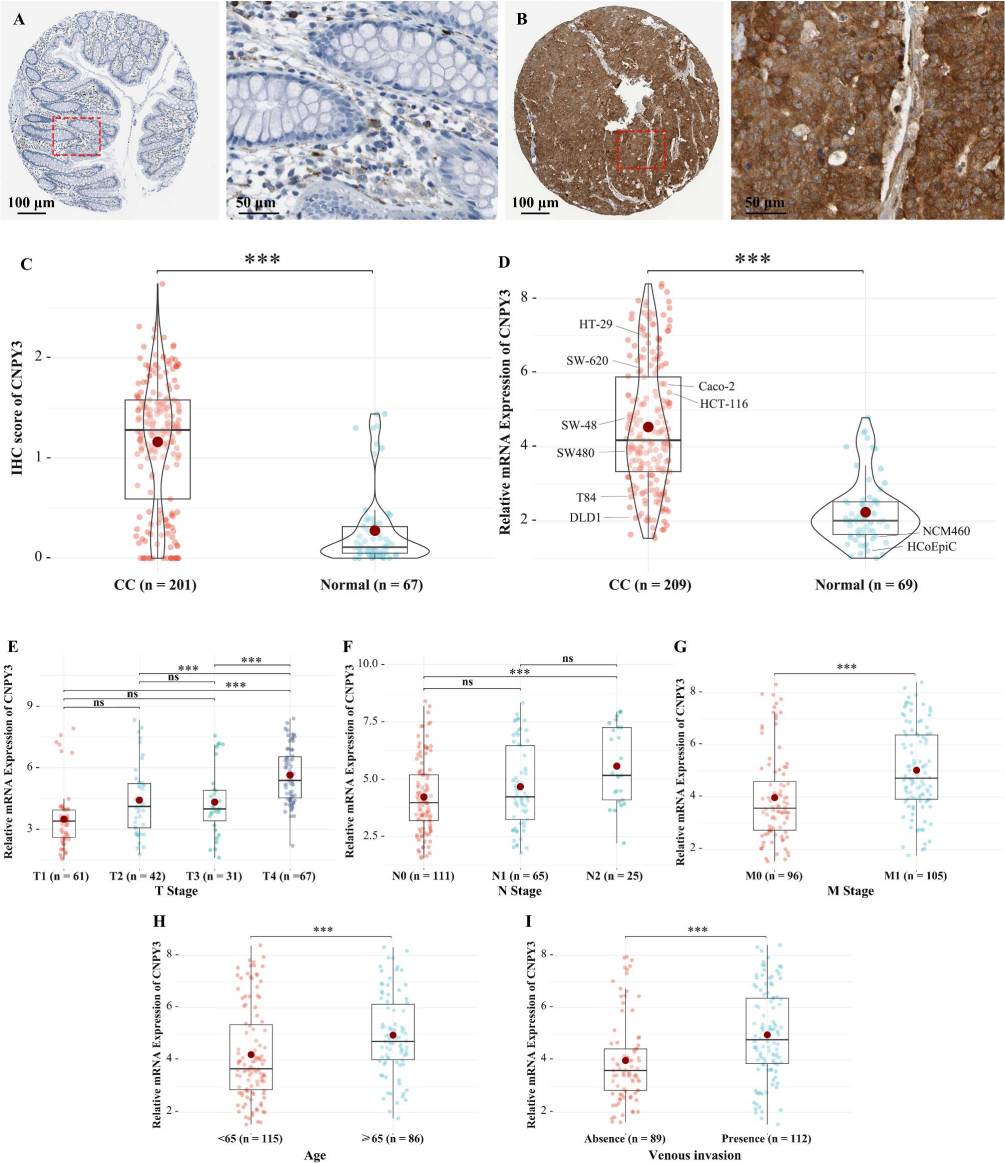
Elevated CNPY3 expression in colon cancer tissues and cell lines. (**A**) Representative Immunohistochemical staining images for normal tissue. (**B**) Representative Immunohistochemical staining images for tumor tissue. (**C**) Quantitative IHC analysis revealed significantly higher CNPY3 scores in colon cancer tissues compared to adjacent normal tissues. (**D**) qRT-PCR results confirmed elevated CNPY3 mRNA levels in colon cancer samples and cell lines compared to controls. (**E**) Correlations between CNPY3 expression and T stage. (**F**) Correlations between CNPY3 expression and N stage. (**G**) Correlations between CNPY3 expression and M stage. (**H**) Correlations between CNPY3 expression and Age. (I) Correlations between CNPY3 expression and Venous invasion. * *P* < 0.05, ** *P* < 0.01, *** *P* < 0.001

The IHC analysis indicated that CNPY3 was predominantly localized within glandular structures and vascular smooth muscle cells in noncancerous tissue areas. Representative images are provided in Fig. 1A and B. Quantitative analysis of CNPY3 IHC scores, which combine staining intensity and percentage of stained cells, revealed a notably higher mean score in CC tissues (mean score: 1.16) compared to adjacent normal samples (mean score: 0.28) (Fig. 1C). The qRT-PCR analysis confirmed a significant elevation of CNPY3 mRNA in CC samples relative to controls (Fig. 1D). Among eight CC cell lines and two normal colonic epithelial cell lines, the HT-29 and SW-620 cell lines showed the highest expression of CNPY3, making them ideal for subsequent gene knockdown experiments. Bioinformatic analyses from the TCGA (Figure S1A) and GTEx databases (Figure S1B) consistently demonstrated higher CNPY3 levels in CC tissues compared to normal tissues. In addition, CNPY3 overexpression was significantly associated with advanced T stage (Fig. 1E), N (Fig. 1F), and M stages (Fig. 1G), older patient age (Fig. 1H), and presence of venous invasion (Fig. 1I).

### Correlation of CNPY3 expression with clinicopathological parameters and patient prognosis

**Fig. 2:**
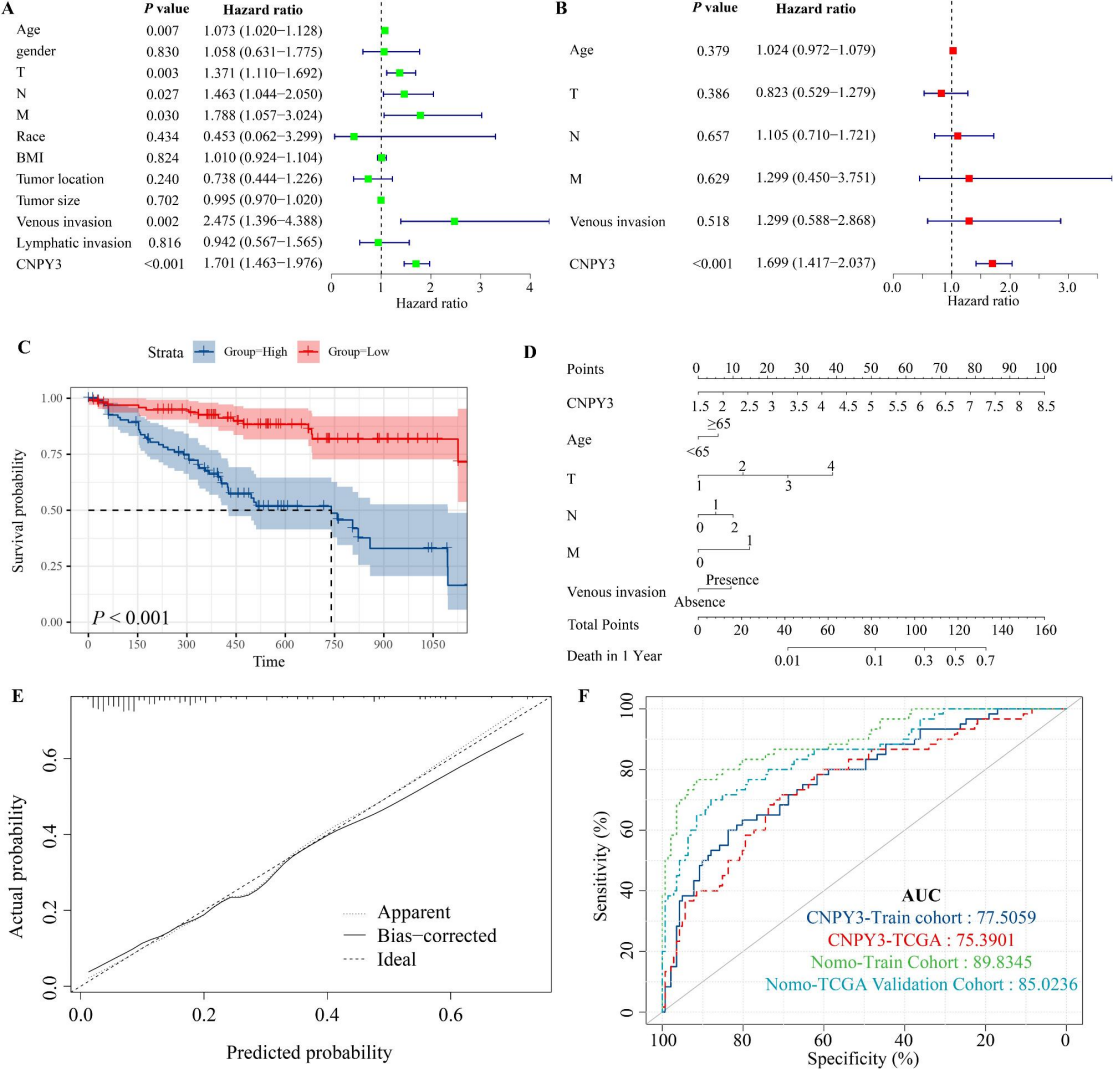
Correlation of CNPY3 expression with clinicopathological parameters and patient prognosis. (**A**) Univariate Cox regression analysis identified several clinicopathological features, including elevated CNPY3, as predictors of poor prognosis. (**B**) Multivariate Cox analysis highlighted high CNPY3 and advanced T stage as independent indicators of unfavorable outcomes. (**C**) Kaplan-Meier survival curve showed reduced overall survival for high CNPY3 expression patients. (**D**) Nomogram for predicting 1-year mortality risk. (**E**) Calibration curve of the nomogram. (**F**) ROC analysis for CNPY3 and nomogram model’s accuracy

Patients were stratified into high- and low-CNPY3 expression groups using a median cutoff value of 4.18, with corresponding clinicopathological features detailed in Supplementary Table S2. Univariate Cox regression analysis (Fig. 2A) identified age, T stage, N stage, M stage, venous invasion, and elevated CNPY3 levels as predictors of poor prognosis. Multivariate analysis (Fig. 2B) further identified high CNPY3 expression (HR: 1.699) and advanced T stage (HR: 1.423) as independent indicators of unfavorable outcomes. Kaplan-Meier survival analysis demonstrated a shorter overall survival time for patients with high CNPY3 expression (Fig. 2C). A nomogram was constructed to predict 1-year death probability. As shown in Fig. 2D, the equation of each variable as follows: total point = CNPY3 (14.285714286 * expression level − 21.428571429) + Age (< 65 = 0, 65 and above = 27.72299) + T stage (12.93260 * Stage) + N stage (5.026392 * Stage) + M stage (14.83304 * Stage) + Venous invasion (Absence = 9.464675). Then, the risk of 1-year death could be calculated by the following equation: Risk = -3e-07 * points ^3 + 0.000182611 * points ^2 + -0.016820651 * points + 0.415110348. The calibration curves for the nomogram (Fig. 2E) demonstrated that the model’s performance (the solid line) aligns closely with the ideal model (the diagonal dotted line). The ROC curve was used to assess the accuracy of the nomogram model (Fig. 2F). The results indicated that the AUC of CNPY3 is 0.77 in our clinical cohort and 0.75 in the TCGA cohort. As for the constructed nomogram model, the AUC is 0.90 in our clinical cohort and 0.85 in the TCGA validation cohort.

### Knockdown of CNPY3 reduced proliferation, migration, and colony formation of CC cells

**Fig. 3:**
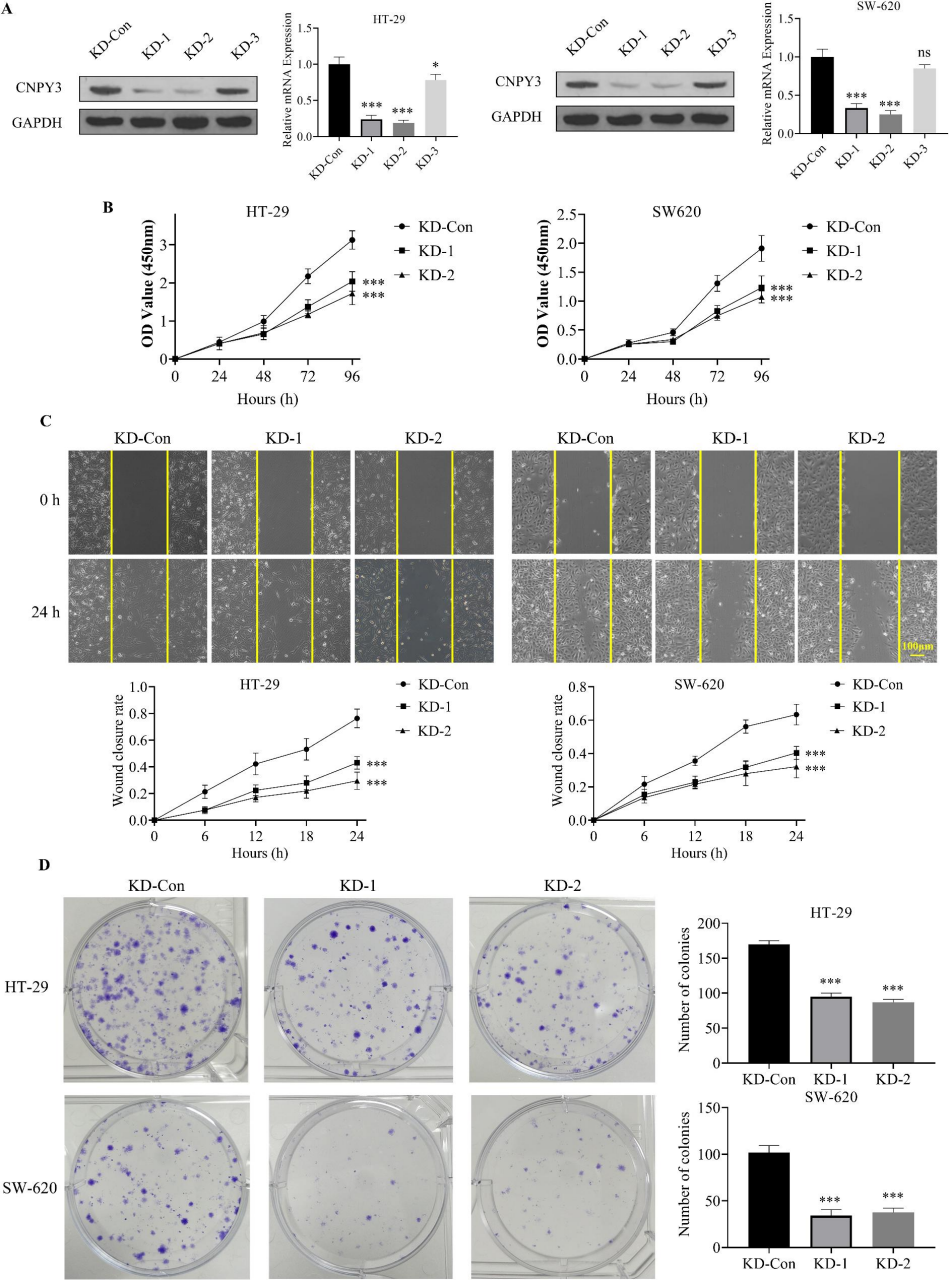
Effects of CNPY3 knockdown on CC cell proliferation, migration, and colony formation. (**A**) Validation of CNPY3 knockdown in HT-29 and SW-620 cells. (**B**) CCK-8 assays showed reduced proliferation in CNPY3-knockdown cells. (**C**) Wound healing assays demonstrated impaired migration post-knockdown. (**D**) Colony formation assays showed decreased colony numbers in knockdown cells. * *P* < 0.05, ** *P* < 0.01, *** *P* < 0.001

Successful CNPY3 knockdown was verified in HT-29 and SW-620 cells, with the KD-1 and KD-2 knockdown groups showing the most effective CNPY3 suppression (Fig. 3A). CCK-8 assays revealed a significant reduction in cell proliferation in both KD-1 and KD-2 cells compared to controls (Fig. 3B). In wound healing assays, migration was substantially impaired in both KD-1 and KD-2 groups, with no significant difference between the two knockdown groups (Fig. 3C). Colony formation assays further illustrated a marked decrease in colony numbers in both HT-29 and SW-620 cells following CNPY3 knockdown, suggesting a critical role for CNPY3 in CC cell survival and proliferation (*P* < 0.05) (Fig. 3D).

### Knockdown of CNPY3 induces cell cycle arrest and enhances apoptosis in CC cells

**Fig. 4:**
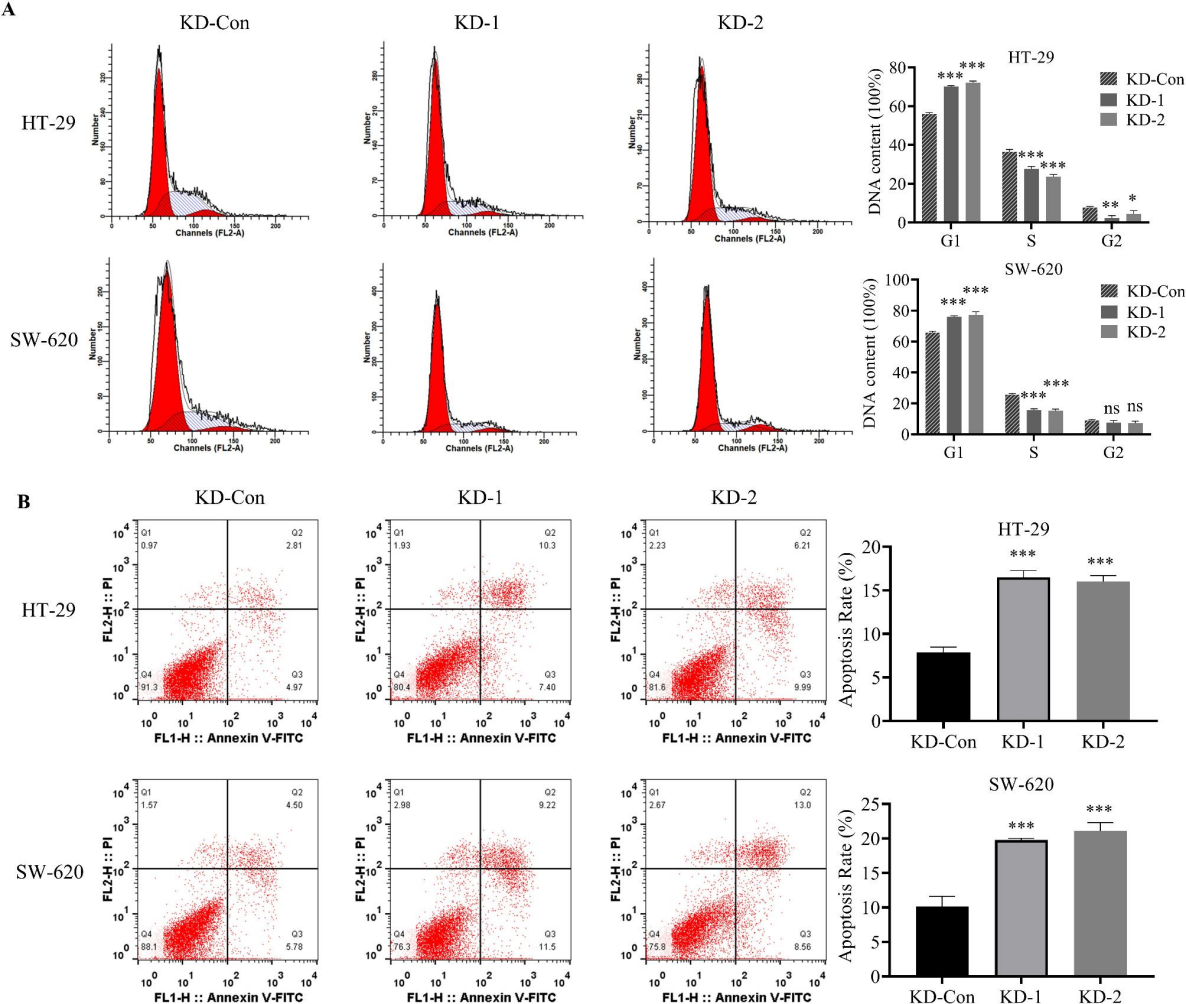
Effects of CNPY3 knockdown on CC cell cycle and apoptosis. (**A**) Cell cycle analysis indicated increased cell death in knockdown cells. (**B**) Apoptosis assays revealed higher levels of apoptotic cells after CNPY3 knockdown. * *P* < 0.05, ** *P* < 0.01, *** *P* < 0.001

Cell cycle analysis revealed that both KD-1 and KD-2 knockdown groups exhibited a significant increase in the sub-G1 population, indicating a notable accumulation of cells undergoing cell death (Fig. 4A). This result suggests that CNPY3 silencing disrupts normal cell cycle progression and promotes cell cycle arrest. In addition, apoptosis assays demonstrated that CNPY3-knockdown cells had significantly higher proportions of apoptotic cells compared to controls (Fig. 4B).

### Role of CNPY3 in regulating the tumor microenvironment in CC

**Fig. 5:**
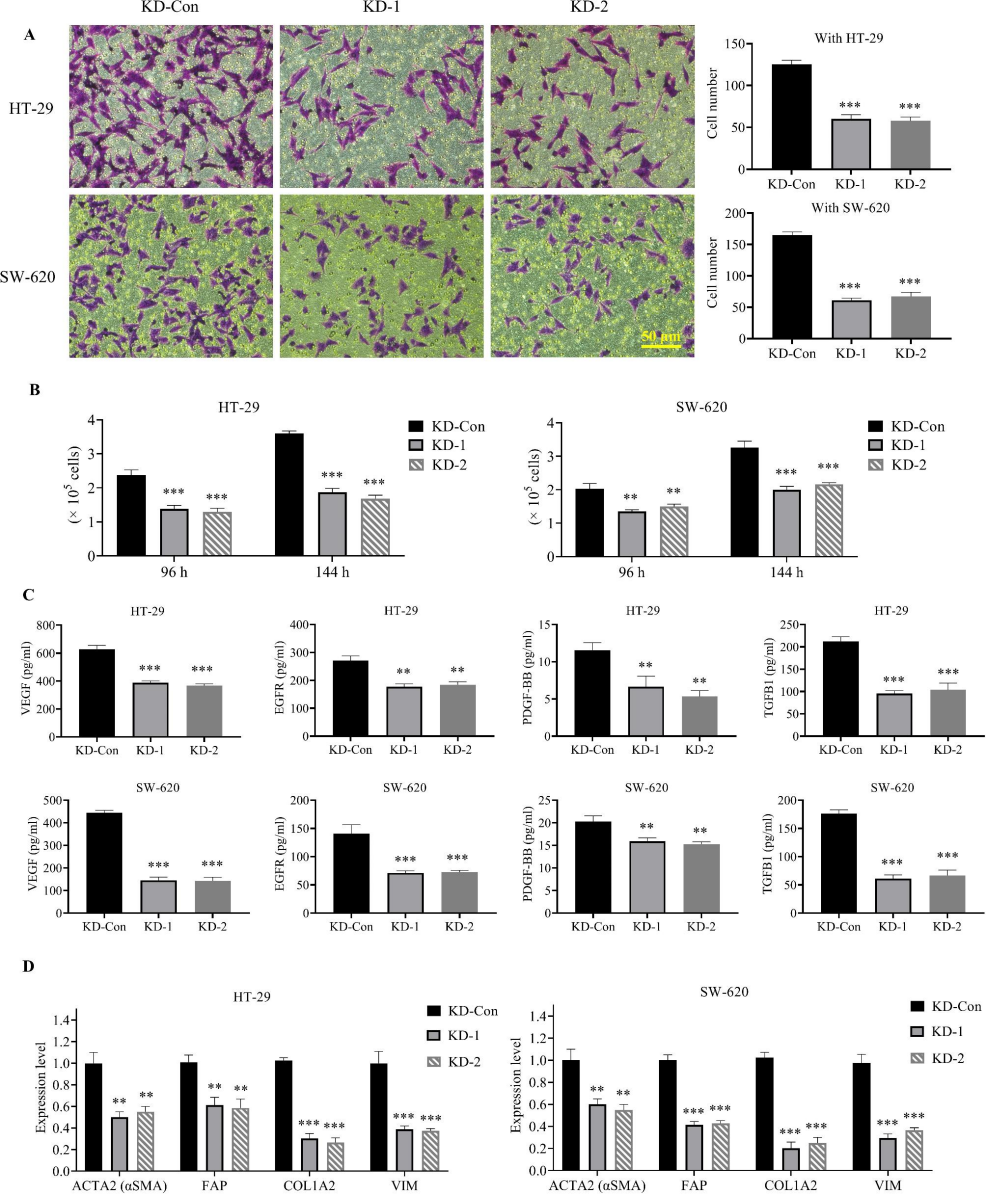
Role of CNPY3 in modulating the tumor microenvironment. (**A**) Co-culture assays indicated decreased fibroblast migration in response to CNPY3-knockdown CC cells. (**B**) Co-culture assays indicated decreased fibroblast proliferation in response to CNPY3-knockdown CC cells. (**C**) Reduced secretion of fibroblast growth factors (EGFR, VEGF, TGFB1, PDGF-BB) in the medium of CNPY3-knockdown cells. (**D**) Suppression of CAF marker expression in fibroblasts co-cultured with CNPY3-knockdown CC cells. * *P* < 0.05, ** *P* < 0.01, *** *P* < 0.001

Co-culture assays demonstrated that fibroblast migration was significantly reduced when fibroblasts were co-cultured with CNPY3-knockdown CC cells (Fig. 5A). Additionally, fibroblast proliferation was inhibited in the presence of CNPY3-knockdown cells from both KD-1 and KD-2 groups (Fig. 5B). Secretion levels of fibroblast growth factors, including EGFR, VEGF, TGFB1, and PDGF-BB, were decreased in the medium of CNPY3-knockdown cells, indicating a potential paracrine effect of CNPY3 on the tumor stroma (Fig. 5C). Furthermore, fibroblasts exposed to CC cells typically undergo conversion to cancer-associated fibroblasts (CAFs), as evidenced by increased CAF markers (e.g., ACTA2, FAP, COL1A2, VIM). However, CNPY3 knockdown inhibited this transformation in both HT-29 and SW-620 cells. (Fig. 5D).

### Altered gene expression profiles in CNPY3-Knockdown CC cells

**Fig. 6:**
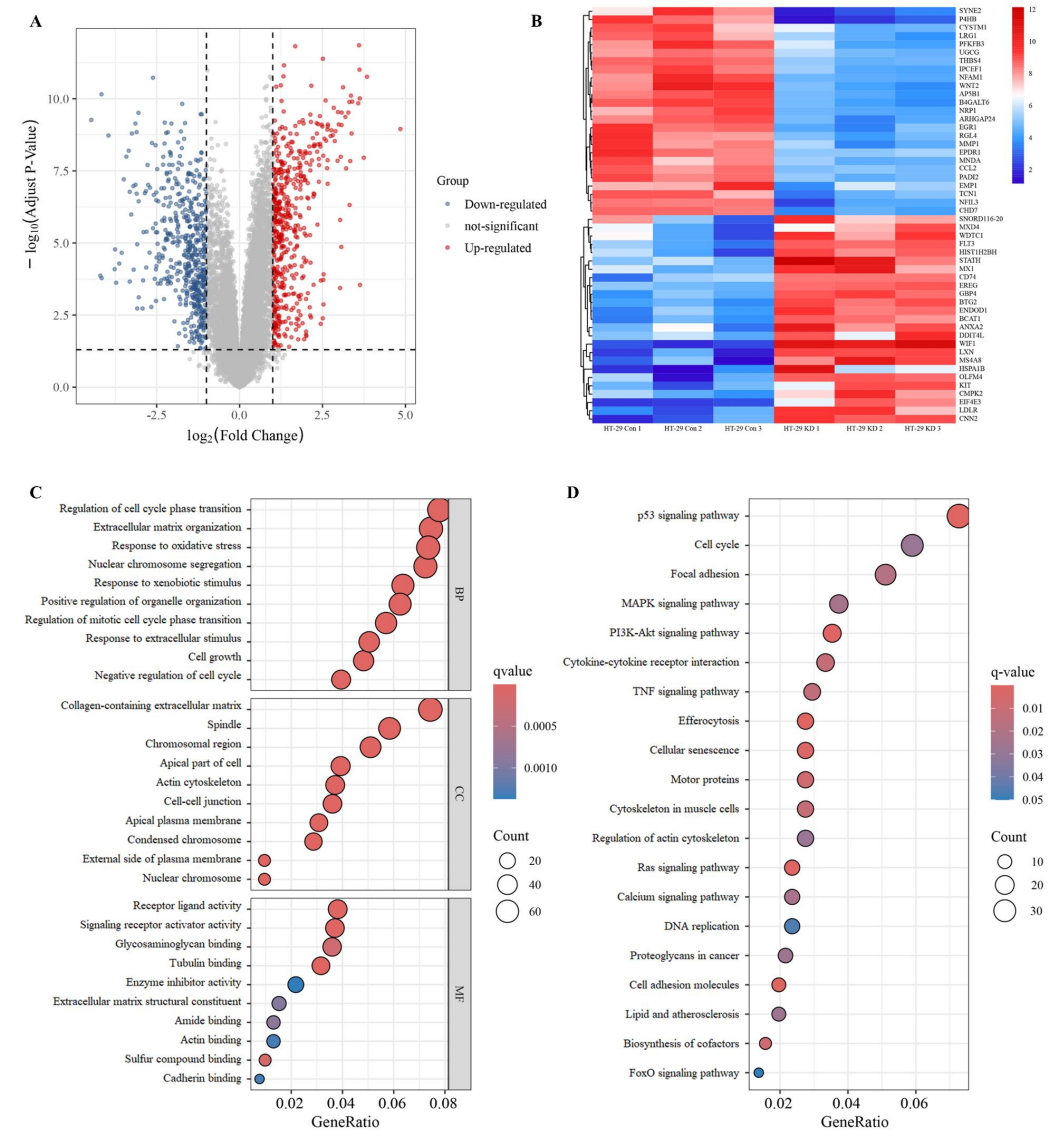
Gene expression alterations in CNPY3-knockdown CC cells. (**A**) Volcano plot of differentially expressed genes in HT-29 cells with CNPY3 knockdown. (**B**) Heatmap of the top 25 upregulated and downregulated genes. (**C**) Gene Ontology (GO) functional annotation analysis. (**D**) Kyoto Encyclopedia of Genes and Genomes (KEGG) Functional annotation

Microarray analysis of HT-29 cells with CNPY3 knockdown identified a total of 1017 differentially expressed genes, with 498 genes upregulated and 519 downregulated (Fig. 6A). The top 25 genes in both categories are displayed in a heatmap (Fig. 6B). Functional annotation revealed significant enrichment in biological processes related to cell cycle progression, extracellular matrix organization, and oxidative stress responses (Fig. 6C). KEGG pathway analysis highlighted the p53 signaling pathway, cell cycle regulation, and focal adhesion as pathways impacted by CNPY3 knockdown (Fig. 6D).

### CNPY3’s influence on tumor-associated pathways and 5-FU sensitivity

**Fig. 7:**
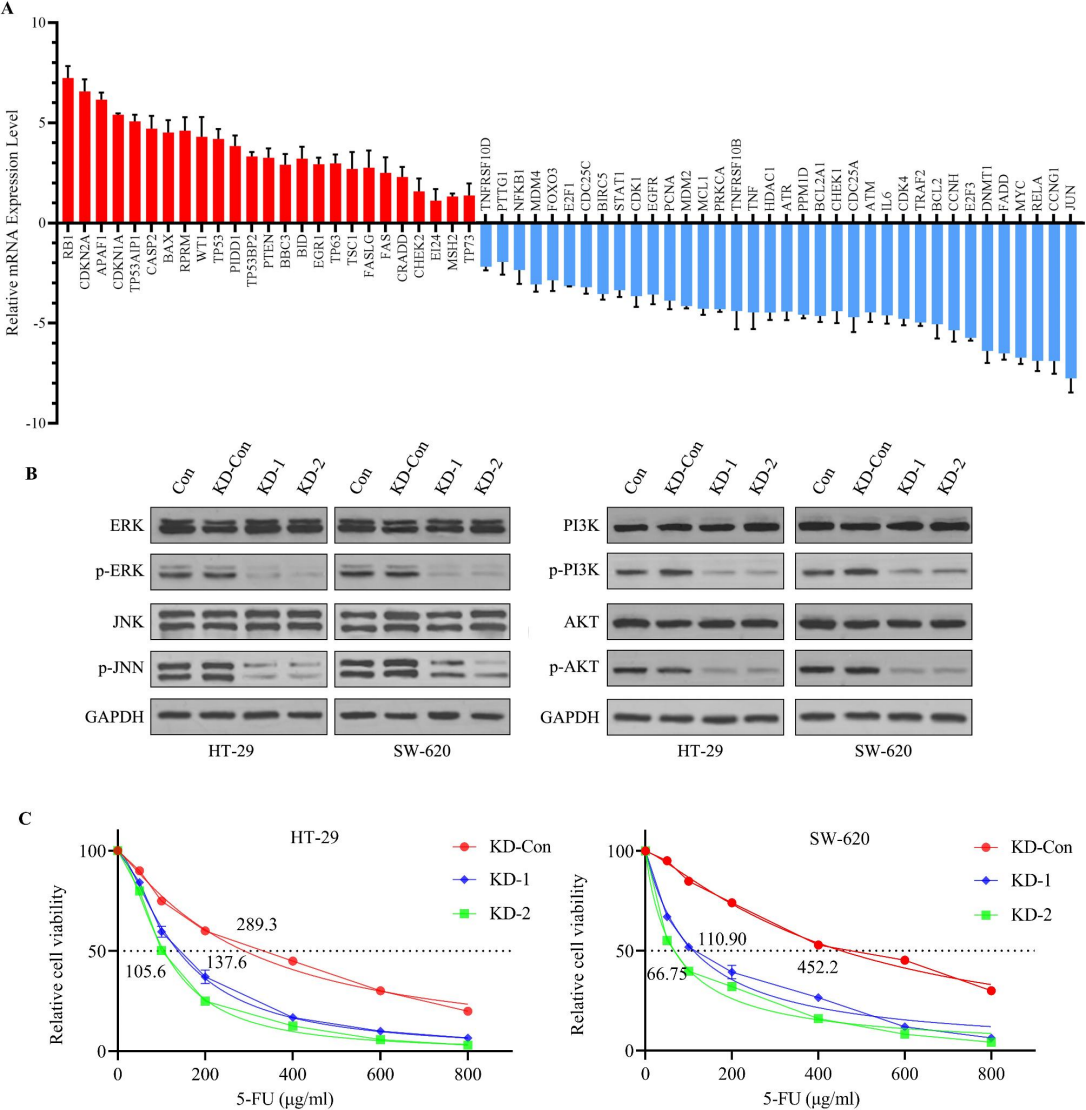
Influence of CNPY3 knockdown on tumor-associated pathways and chemotherapeutic sensitivity. (**A**) Upregulation of p53 pathway-related genes (TP53, FAS, BAX) in CNPY3-knockdown HT-29 cells of the KD-1 group. (**B**) Western blot analysis showed reduced phosphorylation of ERK, JNK, PI3K, and AKT in knockdown cells. (**C**) CCK-8 assay indicated increased sensitivity to 5-fluorouracil, with a lower IC50 in CNPY3-knockdown CC cells

KEGG pathway analysis pinpointed the p53 signaling pathway as a major pathway affected by CNPY3 knockdown. Notably, genes such as TP53, FAS, and BAX were significantly upregulated in CNPY3-knockdown HT-29 cells of the KD-1 group (Fig. 7A), with similar trends observed in the KD-2 group (Figure S2). Other critical pathways, including MAPK and PI3K/AKT, were also influenced, with western blot analysis showing a decrease in phosphorylated ERK, JNK, PI3K, and AKT proteins in knockdown cells (Fig. 7B). Moreover, CCK-8 assays revealed a lowered IC50 for 5-fluorouracil in CNPY3-knockdown CC cells, suggesting enhanced sensitivity to this chemotherapeutic agent (Fig. 7C).

### In vivo impact of CNPY3 knockdown on tumor progression and 5-FU sensitivity

**Fig. 8:**
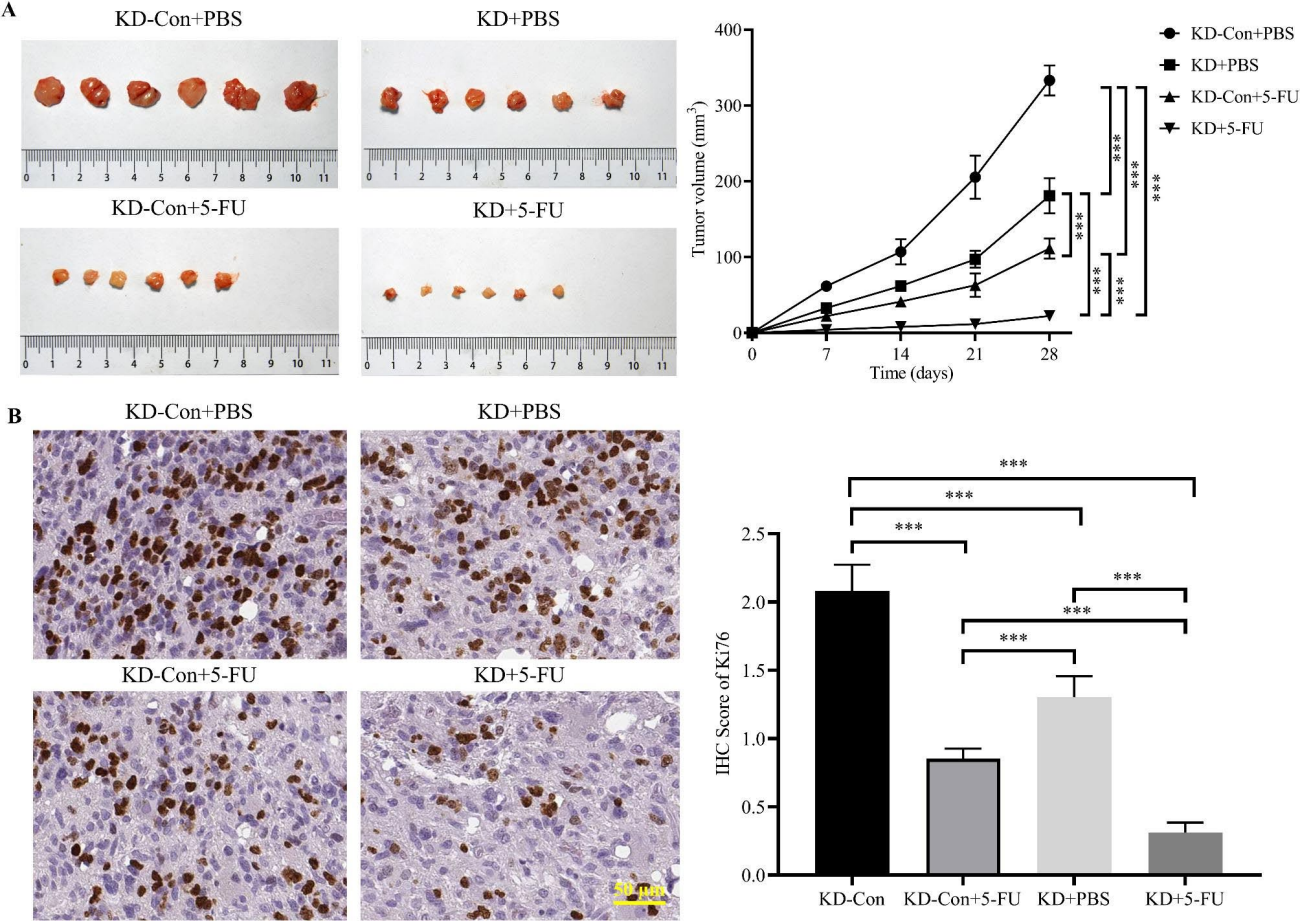
In Vivo Impact of CNPY3 Knockdown on Tumor Progression and 5-FU Sensitivity. (**A**) Images of tumors harvested from xenograft models with different treatment conditions and tumor growth curves. (**B**) Immunohistochemical staining of Ki-67 in tumor tissues from each group and quantification of IHC scores. * *P* < 0.05, ** *P* < 0.01, *** *P* < 0.001

To assess the in vivo impact of CNPY3 knockdown on tumor progression and 5-FU sensitivity, mice were divided into four groups: KD-Con + PBS, KD + PBS, KD-Con + 5-FU, and KD + 5-FU. Tumor volumes were measured at various time points (days 7, 14, 21, and 28). As shown in Fig. 8A, tumors in the KD + PBS group were significantly smaller than those in the KD-Con + PBS group, suggesting that CNPY3 knockdown significantly affects tumor growth in vivo. In addition, CNPY3 knockdown significantly reduced tumor growth in the KD + 5-FU group compared to other groups, with the smallest tumor volumes observed in this group, demonstrating enhanced sensitivity to 5-FU following CNPY3 knockdown.

Our IHC analysis detected Ki67 expression to assess tumor cell proliferation (Fig. 8B). Tumor tissues from mice in all three experimental groups showed significantly lower Ki67 expression compared to the control group. Notably, the KD + 5-FU group exhibited the lowest Ki67 staining, indicating a marked reduction in tumor cell proliferation. Quantitative analysis revealed that Ki67 expression was reduced by 37.3%, 59.0%, and 85.1% in the KD + PBS, KD-Con + 5-FU, and KD + 5-FU groups, respectively, relative to the KD-Con group.

## Discussion

In this study, we examined the expression and functional role of CNPY3 in CC. Immunohistochemistry and qRT-PCR analyses revealed significantly higher CNPY3 expression in CC tissues and cell lines compared to normal controls. Elevated CNPY3 levels were correlated with advanced tumor stages, older patient age, and poor prognosis. Functional studies demonstrated that CNPY3 knockdown in CC cell lines (HT-29 and SW-620) led to reduced proliferation, migration, and colony formation, alongside increased apoptosis. To ensure the robustness of our findings, two independent knockdown groups were used to validate the in vitro experiments. Additionally, CNPY3 knockdown inhibited fibroblast proliferation and migration in co-culture experiments and suppressed the transformation of fibroblasts into CAFs. Gene expression profiling indicated significant changes in pathways related to the cell cycle and extracellular matrix organization, with key tumor-related pathways, including p53, MAPK, and PI3K/AKT, being affected by CNPY3 knockdown. In vitro and in vivo experiments further validated that CNPY3 knockdown enhanced CC cell sensitivity to 5-fluorouracil, as evidenced by reduced IC50 values in cell viability assays and enhanced tumor growth suppression in 5-FU-treated xenograft models. In vivo experiments also confirmed that CNPY3 knockdown significantly reduced tumor growth in mice. Collectively, these findings suggest that CNPY3 plays a critical role in promoting CC progression and modulating the tumor microenvironment, potentially serving as a prognostic biomarker and therapeutic target.

Previous studies have identified CNPY3 as an important regulator in various cancers, exhibiting multifaceted roles in cancer progression and response to treatment. For instance, CNPY3 has been implicated in prostate cancer, where it serves as a direct target for gambogic acid to induce pyroptosis by altering its lysine lactylation and promoting lysosomal rupture (Zhang et al. 2024). In gastric cancer, CNPY3 enhances liver metastasis by forming a regulatory axis with SLITRK4, which drives endocytosis and recycling of the TrkB receptor, ultimately fostering metastatic potential (Zhou et al. 2023). Additionally, in macrophages within the tumor microenvironment, CNPY3 is essential for TLR2 trafficking, playing a crucial role in preventing phospholipid peroxidation-induced resistance to ferroptosis (Luo et al. 2024). These findings underscore CNPY3’s diverse roles in cancer biology, highlighting its potential as a therapeutic target across different tumor types. Building on the established role of CNPY3 in various cancers, our study underscores its significance in colon cancer, drawing parallels to findings in other malignancies.

In IHC and qRT-PCR analysis, we observed that elevated CNPY3 expression in CC correlates with poorer patient outcomes. This finding correlated with our early research (Gao et al. 2024). In this study, our analysis of CNPY3 expression, its clinical correlation, and prognostic impact is based majorly on clinical samples from a Chinese population. In contrast, our previous study relied on bioinformatic data from the TCGA cohort. In addition, the nomogram constructed from these variables—including CNPY3 expression, age, T stage, N stage, M stage, and venous invasion—demonstrated strong predictive power for 1-year mortality risk. With an AUC of 0.90 in our clinical cohort and 0.85 in the TCGA validation cohort, this model showed high accuracy and robustness across independent datasets, which further strengthens its reliability. The consistency of our findings across these two independent populations strengthens the evidence that elevated CNPY3 expression correlates with poorer outcomes in CC.

Our study delved into the role of CNPY3 in modulating the tumor microenvironment, particularly focusing on its effects on fibroblast behaviors. While CNPY3 has been implicated in cancer progression, its influence on the tumor stroma remains less understood. Our findings reveal that CNPY3 plays a role in altering fibroblast activity through two main mechanisms. First, CNPY3 impacts fibroblast migration by regulating the release of critical cytokines, including EGFR, VEGF, TGF-β1, and PDGF-BB, which are known to drive fibroblast motility (Chen et al. 2024a, b, c). The EGFR signaling pathway, often activated by ligands like epiregulin, has been shown to play a pivotal role in CC progression by promoting cellular proliferation and motility (Cheng et al. 2021). Elevated EREG expression in the tumor microenvironment, particularly from fibroblasts and macrophages, activates EGFR signaling through autocrine and paracrine loops, enhancing fibroblast migration and contributing to therapeutic resistance in CC (Yoshida 2020; Luraghi et al. 2014). VEGF promotes angiogenesis (Ando et al. 2019), while TGF-β1 is well-documented to facilitate fibroblast activation and transformation into CAFs through pathways involving CaMKII and Smad3 (Chen et al. 2022). Second, CNPY3 appears to facilitate the transition of normal fibroblasts into CAFs. Given that CAFs possess enhanced migratory and supportive characteristics compared to normal fibroblasts, this conversion likely aids in creating a more pro-tumorigenic microenvironment (Chen et al. 2024a, b, c; Wang et al. 2024). CAFs actively promote colon cancer cell growth, proliferation, and metastasis through paracrine mechanisms and specific signaling axes (Wei et al. 2024a, b; Li et al. 2024). For instance, CAFs in colon cancer release cartilage oligomeric matrix protein, which enhances cancer cell proliferation and metastasis via activation of the RLIM/PML axis, thus supporting the tumor’s invasive properties (Chen et al. 2024a, b, c; Sánchez-Ramírez et al. 2024). Recent studies have shown that CAFs in CC can inhibit effector T cell function by expressing NECTIN2, which binds to immune-inhibitory receptors and promotes T cell exhaustion (Agorku et al. 2024). This immune-suppressive role of CAFs underscores their potential to shield the tumor from immune attack, thereby facilitating tumor survival and growth (Emon et al. 2024; Chen et al. 2024a, b, c). By promoting fibroblast migration and CAF transformation, CNPY3 may contribute to a dynamic and supportive tumor stroma that accelerates cancer progression.

Another significant finding of this study is that CNPY3 knockdown affects several key tumor-related pathways, particularly the p53, MAPK, and PI3K/AKT pathways. The modulation of these pathways by CNPY3 may have critical implications for 5-FU sensitivity in CC. Research has established that p53 is a crucial tumor suppressor that regulates cell cycle progression and apoptosis, influencing the response to chemotherapy (Yang et al. 2021, 2023). Specifically, studies indicate that 5-FU enhances p53 activation, which in turn promotes apoptosis through alterations in chromatin accessibility and the expression of pro-apoptotic genes (Ekremoglu and Koc 2021). The results suggested that effective targeting of CNPY3 could augment 5-FU-induced p53 activity, potentially leading to improved therapeutic outcomes in CC. Furthermore, the PI3K/AKT pathway is known to play a pivotal role in mediating cell survival and metabolism, and its dysregulation has been linked to 5-FU resistance (Dong et al. 2022). Inhibiting this pathway can disrupt the metabolic reprogramming that allows cancer cells to evade chemotherapy, thereby enhancing their sensitivity to 5-FU (Zhang et al. 2023). The interplay between CNPY3 knockdown, these critical signaling pathways, and 5-FU responsiveness underscores the potential of CNPY3 as a promising therapeutic target.

This study has several limitations that should be acknowledged. First, the sample size, although sufficient for preliminary analysis, may not provide robust statistical power to detect subtle associations or variations within specific subgroups of CC patients. Second, the study primarily relies on tissue samples from a single institution, which may limit the generalizability of the findings to broader populations. Third, while our study identifies CNPY3’s involvement in several tumor-related pathways, the precise molecular mechanisms remain to be fully elucidated. Despite these limitations, our study simultaneously investigates the role of CNPY3 in both tumor biology and the cancer microenvironment. While many studies focus exclusively on gene function within cancer, our approach highlights the importance of understanding how these genes also impact the surrounding microenvironment. By elucidating the roles of CNPY3 in CC and its microenvironment, our findings pave the way for the development of novel therapeutic strategies targeting both tumor cells and CAFs, ultimately enhancing treatment efficacy.

## Conclusions

In conclusion, CNPY3 is significantly overexpressed in CC tissues, correlating with advanced disease stages and poor prognosis. Functional assays indicate that CNPY3 enhances tumorigenic properties, including cell proliferation and migration, while also influencing the tumor microenvironment and fibroblast behaviors. Moreover, CNPY3 knockdown increases sensitivity to 5-fluorouracil, highlighting its potential as a therapeutic target. These findings emphasize the critical role of CNPY3 in CC progression and its promise for future clinical applications.

## Electronic supplementary material

Below is the link to the electronic supplementary material.

Supplementary Material 1

Supplementary Material 2

Supplementary Material 3

Supplementary Material 4

## Data Availability

No datasets were generated or analysed during the current study.

## Abbreviations

CNPY3: Canopy FGF signaling regulator 3
CC: Colon cancer
CAFs: Cancer-associated fibroblasts
DAB: Diaminobenzidine
IHS: Immunohistochemistry scores
COAD: Colon adenocarcinoma
TCGA: The Cancer Genome Atlas
GEPIA: Gene Expression Profiling Interactive Analysis
GO: Gene Ontology
KEGG: Kyoto Encyclopedia of Genes and Genomes
cDNA: complementary DNA
OS: Overall survival
AUC: Area under the curve
IC50: Half-maximal inhibitory concentration
ECL: Enhanced chemiluminescence
qRT-PCR: quantitative real-time PCR
