# Supplementary material for: CNPY3’s regulation of tumor microenvironment and its impact on colon cancer aggressiveness
Source: Mol Med. 2025 Mar 7;31:89. doi: 10.1186/s10020-025-01145-1 (PMC11887163; doi:10.1186/s10020-025-01145-1)
Supplement: Supplementary file 1 — Supplementary Material 1 [file 10020_2025_1145_MOESM1_ESM.docx]

| Supplementary Table 1: Primer sequences | | |
| --- | --- | --- |
|  | Forward | Reverse |
| RB1 | 5'-GCTGCTGTGACTTCTGCTGT-3' | 5'-CTCAGCTGATCCTGGTGACT-3' |
| CDKN2A | 5'-GCCACACGACTGCTGAGTCT-3' | 5'-GTTGCTGCTCCCTTCTCCAA-3' |
| APAF1 | 5'-CCAGTACCCAACTCTGACCA-3' | 5'-GCCAGAGAGTCCTTCACAAA-3' |
| CDKN1A | 5'-ATGCTGAGCAGCTGACAG-3' | 5'-GAGGTTGAGCAGTGTCTC-3' |
| TP53AIP1 | 5'-AGCCAGTCTGTCCTCCTCCT-3' | 5'-GGTCTGTTGGTCTTGGTGTT-3' |
| CASP2 | 5'-GCAAGGAGAAAGCCTGTTGA-3' | 5'-CCATCAGTGGTGCTCAGTAG-3' |
| BAX | 5'-TGCTGAGTACCTGGTCTGAA-3' | 5'-CCTTCTTGGGTCTCCTCCTT-3' |
| RPRM | 5'-ATGGGACGACCCGTTAGTG-3' | 5'-CCAGGATCCTTCAGTCTTG-3' |
| WT1 | 5'-CTGTCCTGGCCGATGTG-3' | 5'-AGCCTGTCGTTGGTGTTG-3' |
| TP53 | 5'-GACCTGACCTCGACCTCTG-3' | 5'-GCGTTTCTTGCTGGTGACAG-3' |
| PIDD1 | 5'-TGAGGCCAGGATGAGGTG-3' | 5'-CTCCACCCAGGAGCAGTAC-3' |
| TP53BP2 | 5'-GGTGAGTGTAGGTCTGCTGA-3' | 5'-TCTGAGGCTTCTTCTGCTTT-3' |
| PTEN | 5'-ATGGGCTTGTAGGAGTGTCC-3' | 5'-TCAGCTGAGGATGCTGAAGG-3' |
| BBC3 | 5'-AGTCTGGGACGAGGCTGAG-3' | 5'-TCTGGTGGTCCAGGTTGGA-3' |
| EGR1 | 5'-GCTCGCTCAGAGGACCTGA-3' | 5'-AGGAGCCATGCGATGGTTA-3' |
| BID | 5'-AGCTGACCAACGTTTCAGC-3' | 5'-GTTGCTGTGTTGGTGTTGA-3' |
| TP63 | 5'-TGGCTGAGGTCGTCATCCT-3' | 5'-CAGTTCTTCAGTGAGTGGG-3' |
| TSC1 | 5'-AGCTGAGCAGAGCCAGAGC-3' | 5'-CAGCTGTCAGCTGTCTGGC-3' |
| FASLG | 5'-AGCTGCTGCTGAGCAGATC-3' | 5'-TGTGCTGACGTCCCTTCTC-3' |
| FAS | 5'-ACAGCAGGTCAGGAGCTTCA-3' | 5'-CTCCGTTGTTCCAGGTGTGA-3' |
| CRADD | 5'-TGGGCTGCCAACTTCAGAA-3' | 5'-GCTGTCACCTCCTTCTGGA-3' |
| CHEK2 | 5'-TCCAGCTCTCCCTCTCCTTG-3' | 5'-TGCCATCCAGTCTCCGTTTC-3' |
| EI24 | 5'-AGGACGACGTGGTGACG-3' | 5'-GGTGTTGTTCTCAGCGGTG-3' |
| MSH2 | 5'-CAGGCTCAGCTGGTCTGTG-3' | 5'-AGGAGGTCAGGACAGGTCAC-3' |
| TP73 | 5'-ATCCGTCGGTGCTGGTTC-3' | 5'-GCTGACCCAGCAGGTTGT-3' |
| TNFRSF10D | 5'-CAGGACCTGAGGAGGAGGAC-3' | 5'-TGCTTGTGCTCTTCTCCTG-3' |
| PTTG1 | 5'-CAGCGCAGCTGTTGGTGTC-3' | 5'-CTGTTCTCCTCGTCCTCCTG-3' |
| NFKB1 | 5'-GACCCGCTCCTCCACAT-3' | 5'-TCACGTTGTTTCAGGTTG-3' |
| MDM4 | 5'-AAGGAGACCTCGCTCTGCT-3' | 5'-TGCTGCTCTTGTCCTCTGT-3' |
| FOXO3 | 5'-AAGACGACGACTCCGTCAG-3' | 5'-CAGCTCTTCCAGGACCTCC-3' |
| E2F1 | 5'-TGTGCGCCGGTCTCCTT-3' | 5'-CCTTGTTGCTGCCGTTGAT-3' |
| CDC25C | 5'-ACGCTGCTGACTCCGAGC-3' | 5'-TCTGCTGCTGAGCAGCTG-3' |
| BIRC5 | 5'-TGGAAGCCAGCAGACCTG-3' | 5'-TCTTCGTTTCCGCCGTG-3' |
| STAT1 | 5'-GCTGCTGCTGACCTCTCC-3' | 5'-CGCCAGGCCGACTTCTAC-3' |
| CDK1 | 5'-CCTGCTGTGTCCTCGACCT-3' | 5'-AGGACCCTGGAGCCTGAAC-3' |
| EGFR | 5'-ACGCTGCCAGGCTGTTTCT-3' | 5'-TCACCCAGTTCCTGATGGT-3' |
| PCNA | 5'-AAGGAGGACCTCCTGGACC-3' | 5'-AGCAGCCAGGAGGAGATG-3' |
| MDM2 | 5'-GCTGCTGTGAGGAGATGCT-3' | 5'-GAGGCCAGGTTTCACAGT-3' |
| MCL1 | 5'-GTGGAACGAGCTTCATGGA-3' | 5'-CCTTCTCTGAGCCTGTCC-3' |
| PRKCA | 5'-GAGGCTGCTGACCCATGAT-3' | 5'-CCACCTCCCTCGGACAAAT-3' |
| TNFRSF10B | 5'-GAGTGCTGCTCTTGTGAGG-3' | 5'-GAGGCTGCTGTGGTGAGG-3' |
| TNF | 5'-CAGGCTTCTCTGGGATTTC-3' | 5'-AGACAGAGGGGCTGAGGTC-3' |
| HDAC1 | 5'-AGAGGTGCGGTCACACAG-3' | 5'-CTGTCCTTCTCGGAGCAG-3' |
| ATR | 5'-AGCAGCTCCAGGAGAGGA-3' | 5'-TTGGCTGTTCTGGGTTCTC-3' |
| PPM1D | 5'-TGCAGTGTTGGGCTTTGAG-3' | 5'-ACATGCTGTCGCTTCTGTC-3' |
| BCL2A1 | 5'-TGAGCTGGACCTCTGCTG-3' | 5'-CAGGACCTGCTGTTGCTC-3' |
| CHEK1 | 5'-AAGCCTGTGGAATCCAGC-3' | 5'-CGGCGGTTCAGGAAGAA-3' |
| CDC25A | 5'-GCTCCCTGCTTCTTGAGG-3' | 5'-CCTGGTCCAGGGTAGGAA-3' |
| ATM | 5'-GAGGAGTTCAGGATCTCC-3' | 5'-CTCAGCTCTTGAGTGCTG-3' |
| IL6 | 5'-GCCCTGAGAACTGAAAGTGC-3' | 5'-TCTGCTTGTGCAAGGTTGTTG-3' |
| CDK4 | 5'-GCCAGCTGCTGGTCGATG-3' | 5'-GGTAGGAGTGAGGTCGTTG-3' |
| TRAF2 | 5'-AGCTGGGCCAAAGGAGATG-3' | 5'-TGGAGGTCAGGACCCACAC-3' |
| BCL2 | 5'-TGCGTCCACACCTGAGT-3' | 5'-CCCTTCCACCTTCTTCAC-3' |
| CCNH | 5'-GCCGCCGACCTCAGAGG-3' | 5'-CCTGTCATGGACCTCTGTC-3' |
| E2F3 | 5'-AGGCTGGACATCAGGAGG-3' | 5'-CAGGCGGACGCTGTGTA-3' |
| DNMT1 | 5'-AGTGGCTGTGAGGTGAGC-3' | 5'-TGGCTTGCTGTTGAGATG-3' |
| FADD | 5'-GACCCGTCAGGATCCAACTG-3' | 5'-GAGCCTCCACCTCCTGACC-3' |
| MYC | 5'-ATGGGATCTGGGACAGGAC-3' | 5'-CTGCTTCTCCTCCTTGACG-3' |
| RELA | 5'-TTCAGCCTGAGCCCTGGA-3' | 5'-CAGGAGCTCTGCTGAGAGG-3' |
| CCNG1 | 5'-GAGCCAGGAGACCTGCTT-3' | 5'-TGTTGTTGGCAGGCTGGT-3' |
| JUN | 5'-TGCTGAGGACTGCTGCTG-3' | 5'-CAGTTCTGCTTGTGCTTCTG-3' |
