# Supplementary material for: CNPY3’s regulation of tumor microenvironment and its impact on colon cancer aggressiveness
Source: Mol Med. 2025 Mar 7;31:89. doi: 10.1186/s10020-025-01145-1 (PMC11887163; doi:10.1186/s10020-025-01145-1)
Supplement: Supplementary file 2 — Supplementary Material 2 [file 10020_2025_1145_MOESM2_ESM.docx]

| Supplementary Table S2: Clinicopathological data of included patients | | | | |
| --- | --- | --- | --- | --- |
| Variables | All patients (n=201) | CNPY3 high (n=100) | CNPY3 low (n=101) | *P* value |
| Age |  |  |  |  |
| 65> | 115 | 39 | 76 | <0.001 |
| ≥65 | 86 | 61 | 25 |  |
| Sex |  |  |  |  |
| Male | 113 | 54 | 59 | 0.625 |
| Female | 88 | 46 | 42 |  |
| BMI, kg/m^2^ |  |  |  |  |
| 25> | 125 | 61 | 64 | 0.841 |
| ≥25 | 76 | 39 | 37 |  |
| Tumor location |  |  |  |  |
| Right | 112 | 58 | 54 | 0.613 |
| Left | 89 | 42 | 47 |  |
| Tumor size |  |  |  |  |
| 50> | 105 | 50 | 55 | 0.623 |
| ≥50 | 96 | 50 | 46 |  |
| T |  |  |  |  |
| T1+T2 | 103 | 27 | 76 | <0.001 |
| T3+T4 | 98 | 73 | 25 |  |
| N |  |  |  |  |
| N0 | 111 | 47 | 64 | 0.028 |
| N1+N2 | 90 | 53 | 37 |  |
| M |  |  |  |  |
| M0 | 96 | 29 | 67 | <0.001 |
| M1 | 105 | 71 | 34 |  |
| Lymphatic invasion |  |  |  |  |
| Absence | 94 | 44 | 50 | 0.522 |
| Presence | 107 | 56 | 51 |  |
| Venous invasion |  |  |  |  |
| Absence | 89 | 26 | 63 | <0.001 |
| Presence | 112 | 74 | 38 |  |
