# Supplementary figures and images for: CNPY3’s regulation of tumor microenvironment and its impact on colon cancer aggressiveness
Source: Mol Med. 2025 Mar 7;31:89. doi: 10.1186/s10020-025-01145-1 (PMC11887163; doi:10.1186/s10020-025-01145-1)

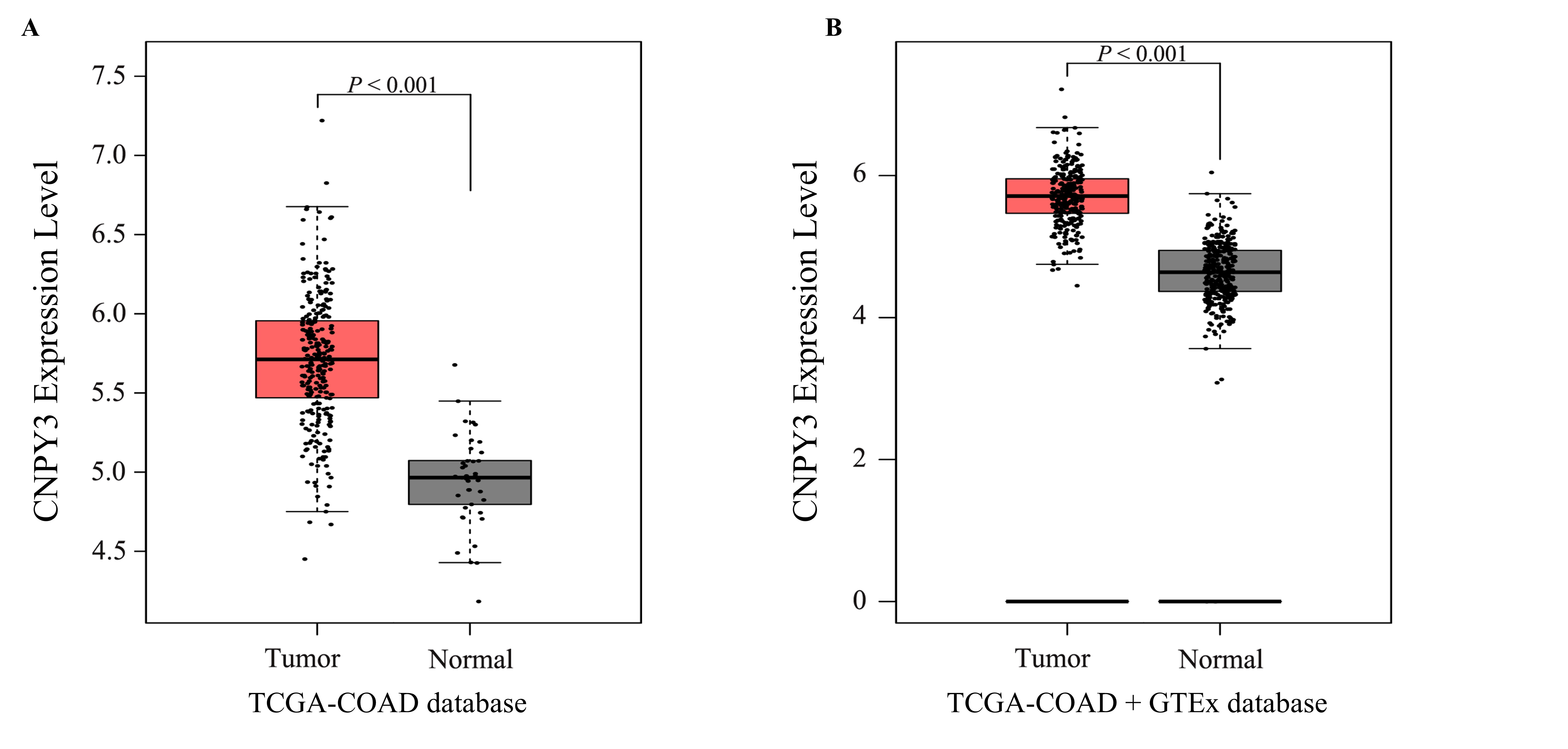

Supplement: Supplementary file 3 — Supplementary Material 3 [file 10020_2025_1145_MOESM3_ESM.jpg]

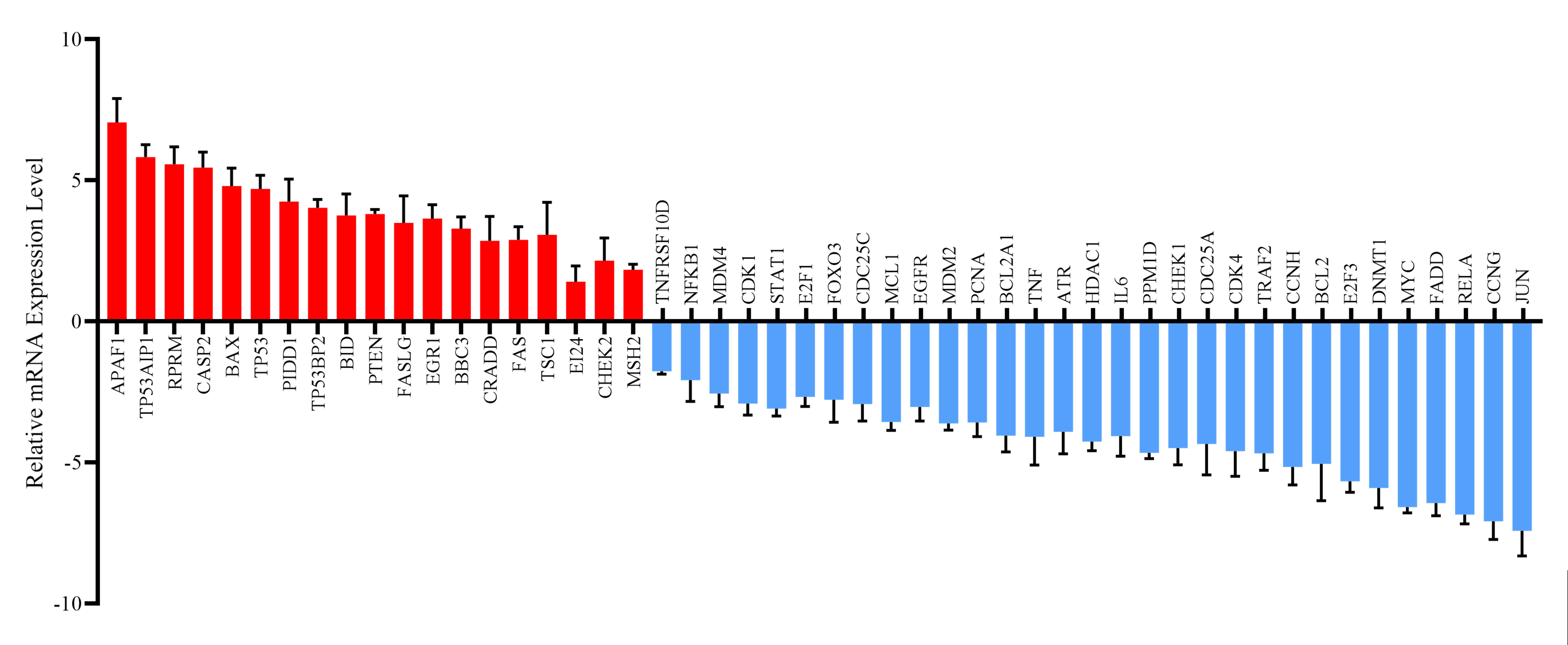

Supplement: Supplementary file 4 — Supplementary Material 4 [file 10020_2025_1145_MOESM4_ESM.jpg]
